# Supplementary material for: Use of Inhaled Corticosteroids and Risk of Acquiring Haemophilus influenzae in Patients with Chronic Obstructive Pulmonary Disease
Source: J Clin Med. 2022 Jun 20;11(12):3539. doi: 10.3390/jcm11123539 (PMC9225538; doi:10.3390/jcm11123539)
Supplement: Supplementary file 1 [file jcm-11-03539-s001.zip › jcm-1768116-supplementary.pdf]

SUPPLEMENARY MATERIAL

Table S1. International classification of Diseases 10th revision (ICD-10) used for the definition of comorbidities in the study population.

Comorbidity	ICD-10 code
Immunodeficiency with predominantly antibody defects	D80
Combined immunodeficiencies	D81
Immunodeficiency associated with other major defects	D82
Common variable immunodeficiency	D83
Other immunodeficiencies	D84
Sarcoidosis	D86
Other disorders involving the immune mechanism, not elsewhere classified	D89
Inflammatory polyarthropathies	M05-M14
Polyarteritis nodosa and related conditions	M30
Other necrotising vasculopathies	M31
Systemic lupus erythematosus (SLE)	M32
Dermatopolymyositis	M33
Systemic sclerosis [scleroderma]	M34
Other systemic involvement of connective tissue	M35
Systemic disorders of connective tissue in diseases classified elsewhere	M36
Myositis	M60
Myocardial infarction	I21-I23
Atrial fibrillation	I48
Heart failure	I110,I130,I132 I42, I50, J81
Hypertension	I10-I15
Peripheral vascular disease	I700-I174,I177,I179
Cerebrovascular disease	I60-I64, G45, G46
Diabetes Mellitus	E10-14
Renal failure	I120, I131, I132; E102, E112, E132, E142 N02-N08, N11, N14, N158; N159, N160, N162-N164,N165 N168, N18, N19, N26, Z992
Asthma	J45
Bronchiectasis	J47
Depression	F32-34

Table S2. Comorbidities at cohort entry in the study population.

Comorbidities	All patients (n=21,218)	*H. influenzae* Positive Patients (n=801)	*H. influenzae* Negative Patients (n=20,417)	P-value
Inflammatory Polyarthropathy	2,053 (9.6)	90 (11.2)	1,963 (9.6)	0.1
Systemic connective tissue Disorder	1,020 (4.8)	35 (4.4)	985 (4.64)	0.6
Myocardial infarction	2,941 (13.9)	110 (13.7)	2,831 (13.9)	0.9
Atrial fibrillation	5,243 (24.7)	223 (27.8)	5,029 (24.6)	0.04
Heart failure	3,658 (17.4)	169 (21.1)	3,489 (17.1)	0.004
Hypertension	9,726 (45.8)	361 (45.1)	9,365 (45.9)	0.7
Renal failure	2,664 (12.6)	123 (15.4)	2,541 (12.6)	0.01
Peripheral vascular disease	3,832 (18.1)	159 (19.9)	3673 (18.0)	0.2

Cerebrovascular disease	3,740 (17.6)	136 (17.0)	3,604 (17.7)	0.6
Diabetes mellitus, type 2	3,739 (17.6)	157 (19.6)	3,582 (17.5)	0.1
Asthma	5,195 (24.5)	270 (33.7)	4,925 (24.1)	<.0001
Bronchiectasis	637 (3.0)	67 (8.4)	570 (2.8)	<.0001
Depression	1,911 (9.0)	79 (9.9)	1832 (9.0)	0.4
Data is reported as n (%)				

Table S3. Months prior to cohort entry in the study population.

	All patients	*H. influenzae* Positive Patients	*H. influenzae* Negative Patients	P-value
Number of patients	21,218 (100)	801 (3.8)	20,417 (96.2)	
Oral corticosteroids				<.0001
No use	13,305 (62.7)	341 (42.6)	12,964 (63.5)	
Low dose	3,947 (18.6)	190 (23.7)	3,757 (18.4)	
High dose	3,696 (18.1)	270 (33.7)	3,696 (18.1)	
Accumulated dose (mg), median (IQR)	750 (250-2000)	750 (500-2500)	625 (250-2000)	<.0001
Long-acting beta2-agonist or long-acting muscarinic-antagonist	14,203 (66.9)	658 (82.2)	13,545 (66.3)	<.0001
Theophylline	696 (3.9)	44 (5.5)	652 (3.2)	.0008
Antibiotics[b]	14,341 (67.6)	654 (81.7)	13,687 (67.0)	<.0001

Data is reported as n (%) or median (IQR), unless indicated otherwise.[a] Low dose < 750 mg; high dose ≥ 750 mg.[b] Any antibiotic drug.

Table S4. Characteristics at cohort entry and according to ICS exposure in 13,324 propensity matched patients with COPD.

	All patients	Patients with high or moderate ICS exposure	Patients with low or no ICS exposure	P-value
Number of patients	13,324 (100)	6,662 (50)	6,662 (50)	
Demographics				
Age (year), median (IQR)	70.2 (62.9-77.7)	70.1 (63.0-77.5)	70.4 (62.7-77.9)	0.6
Age <62	3,019 (22.7)	1,483 (22.3)	1,536 (23.1)	0.5
Age 62-69	3,508 (26.3)	1,823 (27.4)	1,685 (25.3)	
Age 70-77	3,598 (27.0)	1,786 (26.8)	1,812 (27.2)	
Age >77	3,199 (24.0)	1,570 (23.6)	1,629 (24.5)	
Male	5,999 (45.0)	2,986 (44.8)	3,013 (45.2)	0.6
BMI, median (IQR)	25 (21-29)	25 (21-29)	25 (21-29)	.02
BMI-class				0.2
<18.5	1,120 (8.4)	601 (9.0)	519 (7.8)	
18.5-24,9	5,922 (44.5)	2,935 (44.1)	2,987 (44.8)	
25-29,9	3,632 (27.3)	1,822 (27.4)	1,810 (27.2)	
30 -34.9	1,716 (12.9)	841 (12.6)	875 (13.1)	
>35	934 (7.0)	463 (7.0)	471 (7.1)	
Pulmonary parameters				
MRC, median (IQR)	3 (2-4)	3 (2-4)	3 (2-4)	<.0001
FEV_1% predicted, median (IQR)	48 (36-61)	48 (36-60)	48 (36-62)	<.007

FEV_1% predicted, severity of obstruction				.03
≥ 80	517 (3.9)	288 (4.3)	229 (3.4)	
50-79	6,332 (47.5)	3,114 (46.7)	3,218 (48.3)	
30-49	4,804 (36.1)	2,431 (36.5)	2,373 (35.6)	
<30	1,671 (12.5)	829 (12.4)	842 (12.6)	
Active smoking status at study entry	4,416 (33.1)	2,180 (32.7)	2,236 (33.6)	0.3
Hospital-requiring COPD exacerbation 12 months prior to study entry				
0	6,308 (47.3)	3,043 (45.7)	3,265 (49.0)	
1	1,890 (14.2)	928 (13.9)	962 (14.4)	
≥2	5,126 (38.5)	2,691 (40.4)	2,435 (36.6)	
All-cause hospitalisation 12 months prior to study entry (number of patients)	8,684 (65.2)	4,262 (64.0)	4,422 (66.4)	
Comorbidity				
Inflammatory Polyarthropathy	1,318 (9.9)	624 (9.4)	694 (10.4)	
Systemic connective tissue disorder	656 (4.9)	278 (4.2)	378 (5.7)	
Myocardial infarction	1,863 (14.0)	900 (13.5)	963 (14.5)	
Atrial fibrillation	3,419 (25.7)	1,717 (25.8)	1,702 (25.6)	
Heart failure	2,394 (18.0)	1,157 (17.4)	1,237 (18.6)	
Hypertension	6,267 (47.0)	3,109 (46.7)	3,158 (47.4)	
Renal failure	1,712 (12.9)	801 (46.8)	911 (13.7)	
Peripheral vascular disease	2,456 (18.4)	1,191 (17.9)	1,265 (19.0)	
Cerebrovascular disease	2,429 (18.2)	1151 (17.3)	1,278 (19.2)	
Diabetes mellitus, type 2	2,430 (18.2)	1,206 (18.1)	1224 (18.4)	
Asthma	3,443 (25.8)	2,210 (33.2)	1.233 (18.5)	
Bronchiectasis	411 (3.1)	246 (3.4)	165 (2.5)	
Oral corticosteroids [a]				0.5
No use	8,054 (60.5)	3,968 (59.6)	4,086 (61.3)	
Low dose	2,927 (22.0)	1,554 (23.3)	1,373 (20.6)	
High dose	2,343 (17.6)	1,140 (17.1)	1,203 (18.1)	
Other medicine				
Theophylline	419 (3.1)	299 (4.5)	120 (1.8)	<.0001
LABA or LAMA	9,469 (71.1)	5,414 (81.3)	4,055 (60.9)	<.0001
Antibiotics [b]	9,197 (69.0)	4,828 (72.5)	4,369 (65.6)	<.0001
